# A study on the association between tibial plateau fractures and intra-articular soft-tissue injuries under valgus injury mechanisms

**DOI:** 10.1186/s10195-026-00927-5

**Published:** 2026-04-25

**Authors:** Shuo Duan, Shuaishuai Wang, Tongtong Zhu, Qinghan Li, Minglei Zhang

**Affiliations:** Department of Orthopedics, China-Japan Union Hospital of Jilin University, Changchun, 130033 Jilin China

**Keywords:** Tibial plateau fracture, Injury mechanism, Meniscal injury, Ligament injury

## Abstract

**Background:**

While recent investigations have focused on injury mechanism classifications of tibial plateau fractures (TPFs), the association between valgus TPFs and concomitant soft-tissue damage involving menisci and ligaments remains insufficiently elucidated. This study aimed to characterize intra-articular soft-tissue injuries associated with various valgus TPFs and assess the predictive value of lateral plateau depression (LPD) and widening (LPW). Additionally, the analysis extended to other injury mechanisms.

**Materials and methods:**

This study included adult patients with acute tibial plateau fractures who had complete imaging data, excluding patients with open fractures and multiple fractures throughout the body. Imaging examinations were used to assess the fracture mechanism and intra-articular soft-tissue damage.

**Results:**

The study retrospectively analyzed the clinical data of 138 patients with valgus injury TPFs in our hospital. The study compared the incidence of TPFs with intra-articular soft-tissue damage under different injury mechanisms. The result demonstrated that the incidence of valgus hyperextension TPFs combined with medial collateral ligament injuries was relatively high (61.5%) compared with TPFs with other injury mechanism. Multivariable logistic regression and smooth curve fitting revealed significant dose–response relationships of LPD (odds ratio [OR] = 1.408; 95% confidence interval [CI] 1.217, 1.627) and LPW (OR = 1.782; 95% CI 1.387, 2.290) with the risk of lateral meniscus (LM) tears in valgus TPFs. Receiver operating characteristic curves showed the area under the curve (AUC) values and the optimal thresholds of LPD and LPW. For all valgus TPFs, the AUCs of LPD and LPW associated with LM tear were 0.804 (95% CI 0.728, 0.880) and 0.741 (95% CI 0.657, 0.825), respectively. And the optimal threshold for LPD to predict LM tears was 7.11 mm (sensitivity 0.80, specificity 0.74). Subgroup analysis by injury mechanism further demonstrated that, under the valgus extension injury mechanism, the optimal threshold was 8.45 mm (sensitivity 0.68, specificity 0.91). Under the valgus flexion injury mechanism, the optimal threshold was 7.18 mm (sensitivity 0.69, specificity 0.87). Further analyses revealed that varus flexion TPFs demonstrated elevated risks of anterior cruciate ligament injuries (82.5%), lateral collateral ligament injuries (65.0%), and meniscal tears (70.0%), whereas varus hyperextension TPFs showed higher posterior cruciate ligament injury prevalence (56.3%).

**Conclusions:**

LPD serves as a reliable predictor of fractures combined with LM tears. Specifically, under the overall valgus injury mechanism, the possibility of LM tears (particularly the posterior horn tears) should be guarded against when LPD exceeds 7.11 mm. For the valgus extension subtype, the possibility of LM tears (especially the anterior horn tears) should be highly suspected when LPD exceeds 8.45 mm. And for the valgus flexion subtype, an LPD exceeding 7.18 mm should prompt evaluation for LM tears, particularly those affecting the posterior horn.

**Level of evidence:**

Level 3.

**Supplementary Information:**

The online version contains supplementary material available at 10.1186/s10195-026-00927-5.

## Introduction

Tibial plateau fractures (TPFs) constitute complex orthopedic injuries that present substantial clinical management challenges, accounting for approximately 1% of all skeletal fractures [1]. The underlying mechanism predominantly involves excessive loading forces acting on the tibial plateau, typically resulting from a combination of axial compressive forces and coronal plane stresses inducing varus or valgus forces [2]. The knee joint is subjected to concurrent shear and compressive stresses, which elevates the risk of meniscal and ligamentous injuries. Research has shown that the incidence of meniscal and ligament injuries ranges from 39% to 99% and 16.7% to 57%, respectively [3, 4].The presence of intra-articular soft-tissue injuries (menisci and ligaments) frequently complicates the treatment process and directly affects the postoperative functional recovery and long-term prognosis of patients. Early identification and management of intra-articular soft-tissue injuries associated with TPFs are therefore critical for restoring knee joint functionality.

In clinical practice, it is difficult for physicians to detect meniscus and ligament injuries solely through physical examination due to the severe pain and swelling at the fracture site. Therefore, auxiliary examination is the main method for preoperative diagnosis of intra-articular soft-tissue injuries. While magnetic resonance imaging (MRI) remains the main method for assessing intra-articular soft-tissue injuries, its routine application in acute fracture settings remains contentious due to cost constraints, time limitations, and lack of consensus regarding clinical indications [5]. However, numerous studies have demonstrated that X-rays and computed tomography (CT) examinations can also indicate intra-articular soft-tissue injuries in combination with TPFs [6–11]. Existing studies predominantly correlated imaging parameters (lateral plateau depression [LPD], lateral plateau widening [LPW]) with intra-articular soft-tissue injury risks using traditional classification systems such as Schatzker and AO/OTA [12]. However, Schatzker typing and Arbeitsgemeinschaft für Osteosynthesefragen (AO)/Orthopaedic Trauma Association (OTA) typing are mainly based on knee X-rays and are limited by two-dimensional morphology, which makes them difficult to comprehensively assess when it comes to tibial plateau fractures [13–17].

In recent years, research on the correlation between fracture types (based on three-dimensional morphology and injury mechanisms) and soft-tissue injuries have gradually increased. Wang et al. proposed a CT-based categorization of injury mechanisms into four patterns [18]. Firoozabadi et al. [19] further identified a varus hyperextension fracture pattern. Luo et al. proposed a classification system of TPFs based on injury mechanism and initially summarized the soft-tissue characteristics, which attracted extensive academic attention from researchers [2, 20]. Notably, valgus injury mechanisms represent the most prevalent category. Compared with the Schatzker system, the valgus types further refine the classification of lateral TPFs. However, little research has been reported in analyzing the correlation between TPFs and soft-tissue injuries under the valgus injury mechanisms.

This study aims to conduct an in-depth analysis of various valgus TPFs to explore the characteristics and patterns of fracture combined with soft-tissue injuries involving menisci and ligaments. Additionally, we measure LPD and LPW to predict the risk of menisci and ligaments injuries. We expect that this study can provide a potential theoretical basis and new ideas for future research development.

## Materials and methods

### Study design and data sources

This paper is a retrospective study. Patients with TPFs who were treated at our institution between January 2018 and September 2024 were selected as the study subjects. All included patients underwent open reduction internal fixation.

Inclusion criteria included acute-phase TPFs (≤ 3 weeks); age ≥ 18 years; complete knee imaging series including X-ray, CT, and MRI scans; and no history of ipsilateral knee fractures or surgical interventions. All patients completed both MRI (meniscus/ligament assessment) and CT (LPD and LPW measurement).

Exclusion criteria included open or pathological fractures; polytrauma with an Injury Severity Score (ISS) > 16; ipsilateral lower limb fractures except proximal fibula; pre-existing deformities; and significant degenerative changes in the knee joint. Open fractures are often accompanied by high-energy trauma, and the pattern of soft-tissue damage is influenced by confounding factors such as the degree of contamination, which may interfere with the interpretation of biomechanical mechanisms. Secondly, open fractures require emergency debridement and infection control, which limits the timing of imaging assessment.

### Study methods

#### Classification of tibial plateau fracture injury mechanisms

Based on the “Three-Column Theory” proposed by Luo et al. [2], TPFs were classified into six injury mechanism types: varus hyperextension, valgus hyperextension, varus extension, valgus extension, varus flexion, and valgus flexion (Additional file 1: Fig. S1). This classification integrates the initial knee position during injury (hyperextension, extension, or flexion) and the direction of axial force application (varus or valgus).

#### Diagnosis and classification of knee soft-tissue injuries

All patients underwent MRI scanning of the affected knee (Siemens Skyra 3.0 T) using a standard knee MRI protocol, which included proton-density-weighted fat-suppressed sequences in the sagittal, coronal, and axial planes. Meniscal injuries were graded 1–4 based on increased intrasubstance signal intensity [21]. MRI manifestations of grade 3 and 4 were defined as the presence of meniscal tear. MRI manifestations of ligamentous contusion, partial tear, or complete tear were categorized as ligament injury. On MRI, a contusion presents as T2 hyperintensity with intact fiber continuity. A partial tear is characterized by ≥ 50% fiber disruption accompanied by interstitial fluid infiltration. A complete tear demonstrates complete fiber disruption with retracted ends. The soft tissues of the knee joint in this paper are limited to ligaments and meniscus.

#### Measurement of lateral plateau depression and widening

**Fig. 1:**
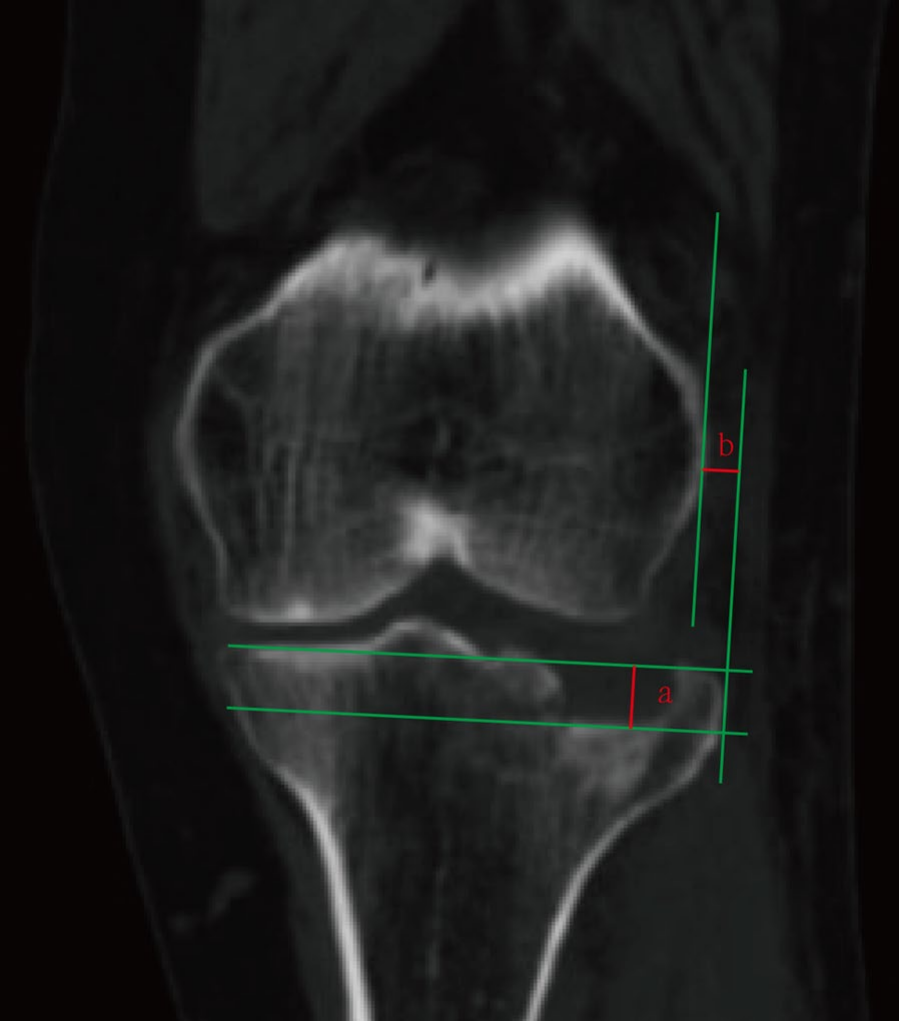
Measurement of lateral plateau depression and widening. Red line A represents LPD, and red line B represents LPW

The measurements of LPD and LPW were performed independently by a senior attending orthopedic surgeon. This evaluator was blinded to the patient’s MRI results during the CT image measurements to ensure objectivity. LPD was defined as the distance between the plateau articular surface tangent line and the lowest point of the lateral plateau collapse. LPW was measured as the distance from the lateral femoral epicondyle tangent to the most distal point of the lateral plateau fracture fragment [10]. When measuring LPD, a tangent line was first drawn along the intact medial tibial plateau. A perpendicular line was then extended from this tangent line to the deepest point of depression in the fractured lateral plateau. The length of this perpendicular line (in millimeters) was recorded as the LPD value, as illustrated in Fig. 1a. For LPW measurement, a line was first drawn perpendicular to the aforementioned tangent line of the medial tibial plateau and tangent to the most distal point of the lateral femoral epicondyle. A second parallel line was then drawn along the outermost edge of the displaced lateral tibial plateau fragment. The distance between these two parallel lines (in millimeters) was defined as the LPW value, as shown in Fig. 1b [11].

### Statistical analysis

All analyses were performed using IBM SPSS 26.0 and R software (version 4.3.0), with statistical significance defined as *P* < 0.05. Categorical variables were expressed as frequencies (%) and analyzed by chi-squared tests. Normally distributed continuous data were reported as mean ± standard deviation and compared using *t*-tests, while non-normally distributed data were presented as median (interquartile range) and assessed using Mann–Whitney *U* tests. Age and sex were adjusted as covariates. Logistic regression analysis and smoothed curve fitting were applied to assess the relationship of LPD and LPW with soft-tissue damage involving menisci and ligaments under valgus injury mechanisms. Receiver operating characteristic (ROC) curves were plotted to determine the optimal cut-off values for LPD and LPW to predict soft-tissue injuries using the maximum value of the Youden index. Predictive performance was assessed by calculating the AUC.

## Results

### Baseline population characteristics

**Fig. 2:**
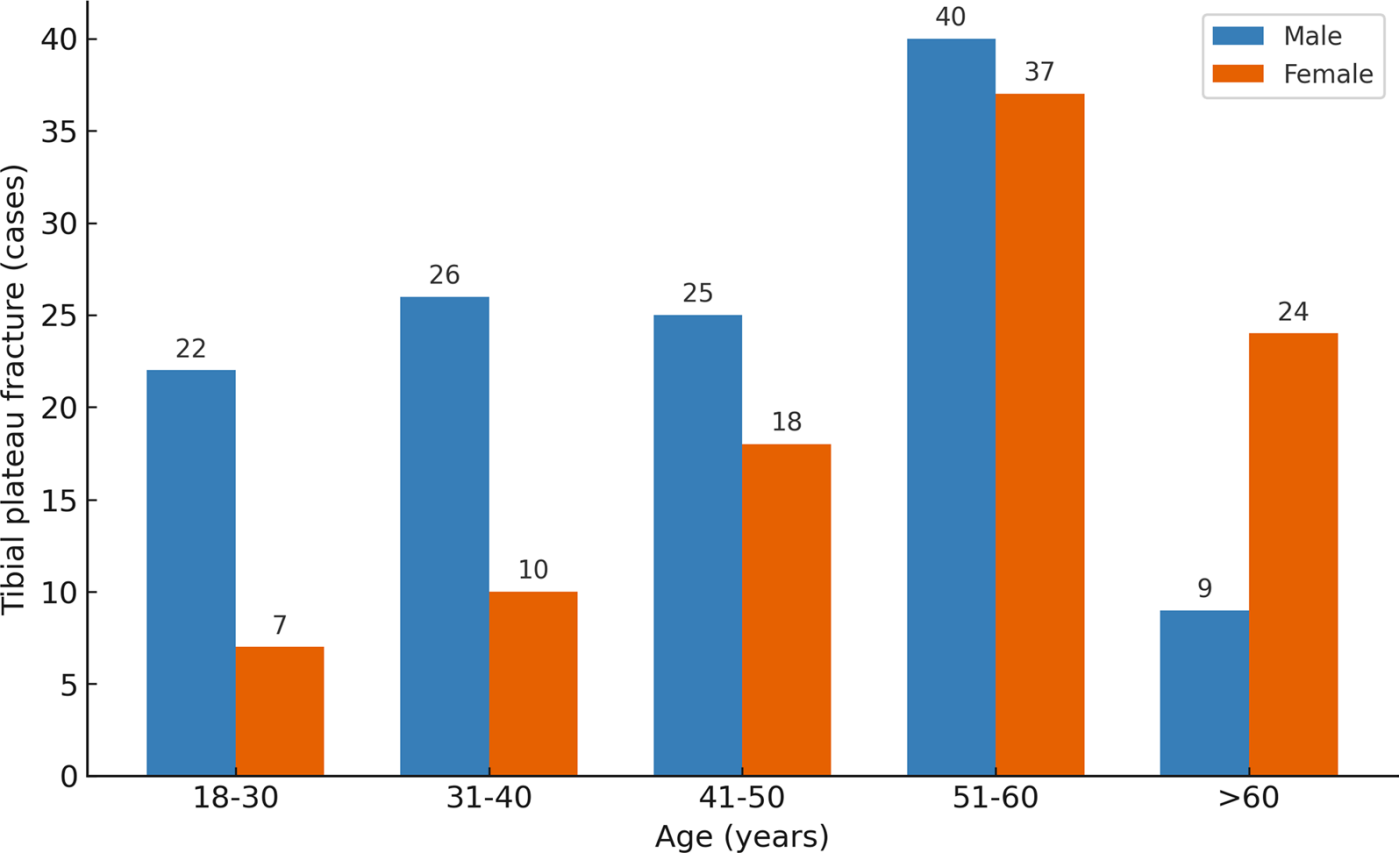
Characteristics of the population distribution of tibial plateau fractures

Based on the predetermined inclusion and exclusion criteria, this study ultimately enrolled 218 patients (Additional file 1: Fig. S2). The cohort consisted of 138 patients with valgus TPFs and 80 cases with other injury mechanisms. A total of 208 patients underwent CT examinations within ≤ 72 h after injury, while MRI scans were completed within the same time frame for 196 patients. All patients underwent the examinations within 3 weeks post-injury and before surgery. The mean age was 47.8 ± 13.3 years. In patients aged < 60 years, male patients accounted for the majority, whereas in those aged ≥ 60 years, female patients constituted the majority (Fig. 2). The sex distribution differed significantly across age groups (*χ*^2^ = 20.130, *P* < 0.001). Patients with meniscal tears were significantly older than those without meniscal tears (Table 1). No differences in age, sex, or side involvement (left/right) were observed among ligament injury subgroups (*P* > 0.05). Based on injury mechanisms, valgus extension type was the most prevalent (31.7%), followed by valgus flexion pattern (25.7%; Additional file 1: Fig. S3). Additionally, meniscal pathology predominantly involved posterior horn tears (Additional file 1: Fig. S4).

**Table 1:** Baseline characteristics of the population

Variable	Total	Meniscus tear	No meniscus tear	*χ*^2^	*P*-value
*Age (years)*					
Continuous variable	47.8 ± 13.3	51.3 ± 1.1	44.1 ± 1.4		< 0.001
*Categorical variable*				18.235	0.001
18–30	29 (13.3)	6 (5.5)	23 (21.3)		
31–40	36 (16.5)	14 (12.7)	22 (20.4)		
41–50	43 (19.7)	22 (20.0)	21 (19.4)		
51–60	77 (35.3)	49 (44.5)	28 (25.9)		
> 60	33 (15.1)	19 (17.3)	14 (13.0)		
*Sex*				2.734	0.098
Male	122 (56.0)	55 (50.0)	67 (62.0)		
Female	96 (44.0)	55 (50.0)	41 (38.0)		
*Laterality*				0.087	0.767
Left	126 (57.8)	62 (56.4)	64 (59.3)		
Right	92 (42.2)	48 (43.6)	44 (40.7)		

### Characteristics of tibial plateau fractures combined with intra-articular soft-tissue injuries under valgus injury mechanisms

This study analyzed 138 valgus TPFs, with valgus hyperextension pattern demonstrating significantly higher incidence of medial collateral ligament (MCL) injuries (*P* < 0.05; Table 2). CT imaging parameters of valgus-type fractures were further evaluated. The median LPD was 6.89 mm (range: 2.11–26.45 mm), and the median LPW was 3.55 mm (range: 1.32–11.25 mm). The lateral meniscus (LM) tear subgroup had significantly greater LPD and LPW values compared with those without LM tears across valgus injury mechanisms (*P* < 0.05; Additional file 1: Table S1).

**Table 2:** Association between TPFs and intra-articular soft-tissue injury under valgus mechanisms

Soft-tissue injury	Total	Injury mechanism			*P*-value
		Valgus hyperextension	Valgus extension	Valgus flexion	
Meniscus tear	67 (48.6)	5 (38.5)	30 (43.5)	32 (57.1)	0.235
LM tear	55 (39.9)	4 (30.8)	25 (36.2)	26 (46.4)	0.400
MM tear	34 (24.6)	4 (30.8)	18 (26.1)	12 (21.4)	0.237
ACL injury	94 (68.1)	10 (76.9)	41 (59.4)	43 (76.8)	0.091
PCL injury	39 (28.3)	7 (53.8)	16 (23.2)	16 (28.6)	0.079
LCL injury	63 (45.7)	7 (53.8)	32 (46.4)	24 (42.9)	0.762
MCL injury	44 (31.9)	8 (61.5)	22 (31.9)	14(25.0)	0.039

ACL, anterior cruciate ligament; LCL, lateral collateral ligament; MM, medial meniscus; PCL, posterior cruciate ligament

**Fig. 3:**
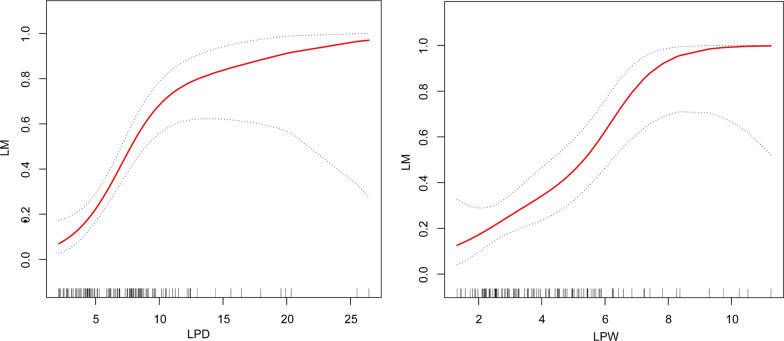
Dose–response associations of LPD and LPW with the risk of LM tear. The solid red line represents the smooth curve fit between variables, and the dashed line indicates the 95% confidence interval. Model adjusted for age and sex

Multivariable logistic regression revealed significant positive associations of LPD (OR = 1.408; 95% CI 1.217, 1.627) and LPW (OR = 1.782; 95% CI 1.387, 2.290) with LM tear risk. This suggested that each unit increase in LPD was associated with a 0.408-fold elevated risk of LM tear, while each unit increase in LPW corresponded to a 0.782-fold increased LM tear risk. Subgroup analysis by injury mechanisms showed that, under the valgus hyperextension pattern, each unit increase in LPD raised LM tear risk by 0.654-fold, and each unit increase in LPW increased the risk by 1.160-fold. For valgus flexion injury mechanisms, each unit increase in LPD and LPW resulted in 0.707-fold and 0.900-fold higher LM tear risks, respectively (Table 3). Additionally, logistic regression further identified positive associations of LPD and LPW with lateral collateral ligament (LCL) injuries, but no significant associations with MCL, anterior cruciate ligament (ACL), or posterior cruciate ligament (PCL) injuries were observed (Additional file 1: Table S2). Smooth curve fitting confirmed dose–response relationships between LPD and LPW and LM tear risk (*P* < 0.05; Fig. 3).

**Table 3:** Logistic regression analysis of LPD and LPW with the risk of lateral meniscus tears

Variable	LM tear		
	OR	95% CI	*P*-value
*Valgus*			
LPD	1.408	1.217, 1.627	< 0.001
LPW	1.782	1.387, 2.290	< 0.001
*Valgus hyperextension*			
LPD	1.141	0.826, 1.577	0.423
LPW	2.621	0.520, 13.204	0.243
*Valgus extension*			
LPD	1.654	1.277, 2.142	< 0.001
LPW	2.160	1.403, 3.324	< 0.001
*Valgus flexion*			
LPD	1.707	1.213, 2.401	0.002
LPW	1.900	1.214, 2.974	0.005

Model adjusted for age and sex

**Fig. 4:**
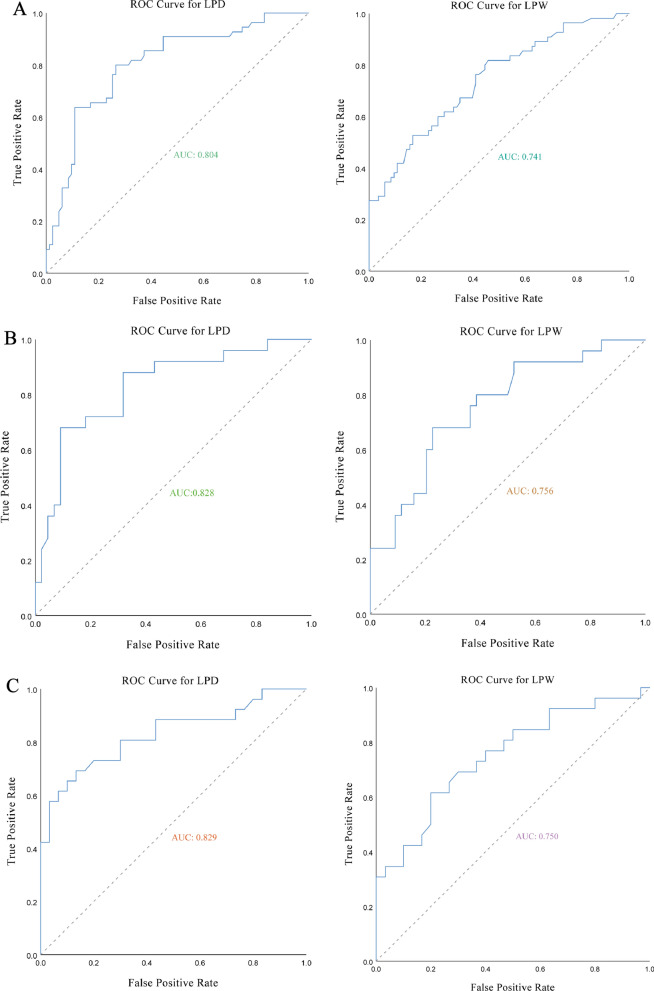
ROC curves of LPD and LPW predicting LM tears under different valgus injury mechanisms. **A** represents all valgus injury mechanisms, **B** represents valgus extension injury mechanism, and **C** represents valgus flexion injury mechanism

**Fig. 5:**
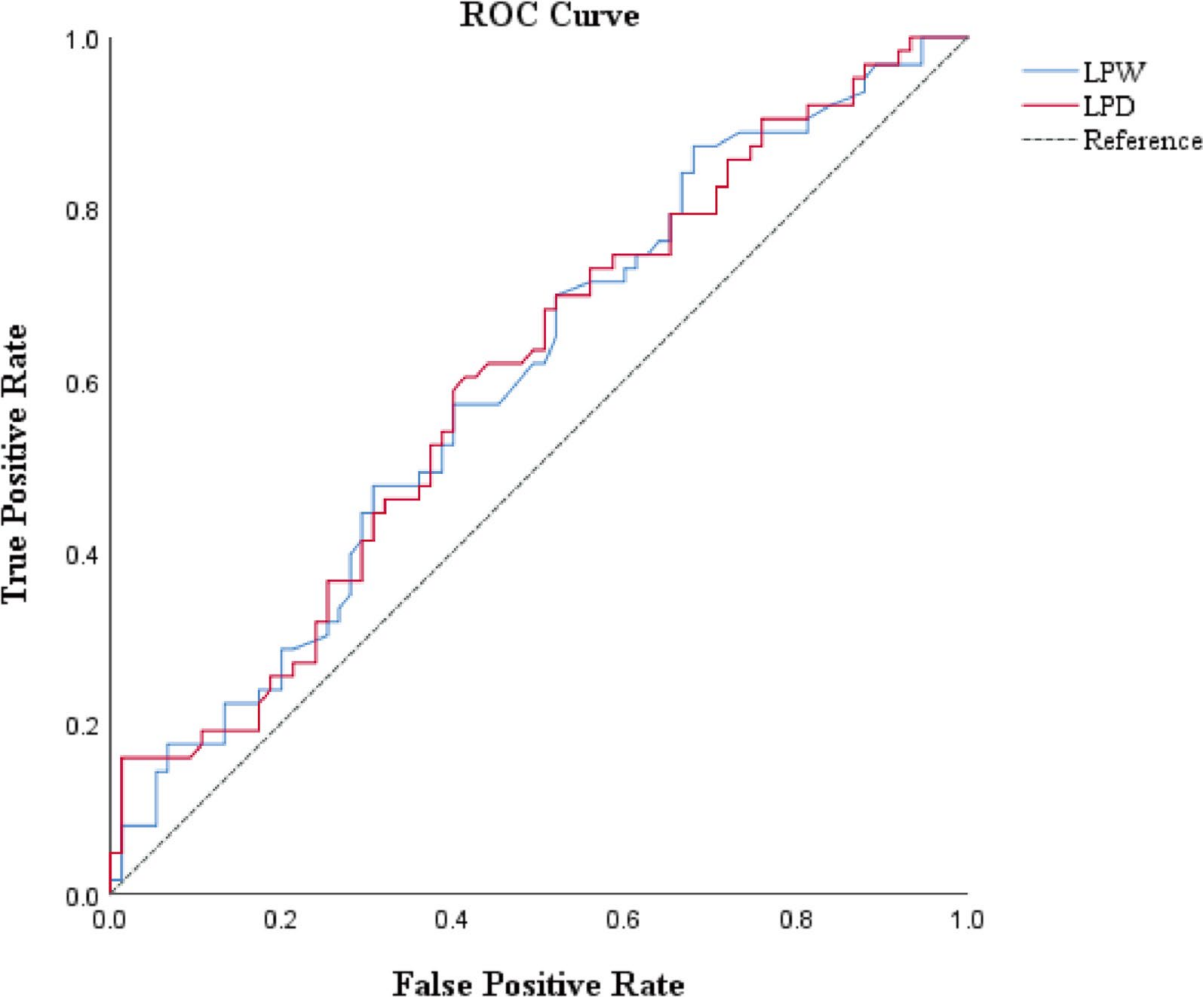
ROC curves of LPD and LPW for predicting LCL injury

ROC curve analysis showed that the AUCs of LPD and LPW associated with LM tear were 0.804 (95% CI 0.728, 0.880) and 0.741 (95% CI 0.657, 0.825), respectively. Under the valgus extension injury mechanism, the AUCs of LPD and LPW were 0.828 (95% CI 0.724, 0.931) and 0.756 (95% CI 0.639, 0.874), respectively. Under the valgus flexion injury mechanism, the AUCs for LPD and LPW were 0.829 (95% CI 0.718, 0.941) and 0.750 (95% CI 0.621, 0.879), respectively (Fig. 4). In contrast, the AUCs of LPD and LPW in predicting LCL injury were 0.603 (95% CI 0.509, 0.697) and 0.600 (95% CI 0.506, 0.694), respectively. This demonstrated that LPD and LPW have limited predictive utility for LCL injury (Fig. 5).

The optimal cut-off values for LPD and LPW in predicting LM tears among patients with valgus TPFs were determined using ROC analysis, with detailed thresholds and corresponding diagnostic performance metrics presented in the table (Table 4).

**Table 4:** Optimal cut-off values of LPD and LPW for predicting LM tears

Variable	Youden index	Optimal cut-off	Sensitivity	Specificity
*Valgus*				
LPD	0.54	7.11	0.80	0.74
LPW	0.36	3.16	0.82	0.54
*Valgus extension*				
LPD	0.59	8.45	0.68	0.91
LPW	0.45	4.88	0.68	0.77
*Valgus flexion*				
LPD	0.56	7.18	0.69	0.87
LPW	0.42	3.28	0.62	0.80

### Correlation of LPD and LPW with LM tear locations under valgus injury mechanisms

Logistic regression revealed persistent positive correlations of LPD and LPW with both anterior and posterior horn LM tears under valgus injury mechanisms, including valgus extension and valgus flexion subgroups (Table 5). ROC curve analysis confirmed that LPD and LPW were reliable predictors of LM anterior and posterior horn tears, with LPD demonstrating superior diagnostic performance (AUC range 0.789–0.843) compared with LPW (AUC range 0.741–0.793; Additional file 1: Table S3, Figs. S5–S7).

**Table 5:** Logistic regression analyses of LPD and LPW with the site of LM tears

Variable	Anterior horn			Posterior horn		
	OR	95% CI	*P*-value	OR	95% CI	*P*-value
*Valgus*						
LPD	1.370	1.189, 1.579	< 0.001	1.350	1.176, 1.551	< 0.001
LPW	1.769	1.378, 2.273	< 0.001	1.655	1.307, 2.097	< 0.001
*Valgus extension*						
LPD	1.556	1.194, 2.026	0.001	1.690	1.177, 2.425	0.004
LPW	1.850	1.225, 2.794	0.003	2.413	1.361, 4.277	0.003
*Valgus flexion*						
LPD	1.481	1.131, 1.939	0.004	1.625	1.172, 2.252	0.004
LPW	1.782	1.221, 2.599	0.003	2.149	1.330, 3.473	0.002

Model adjusted for age and sex

The optimal cut-off values for LPD and LPW in predicting LM anterior or posterior horn tears were summarized in the Tables S4–S6 in Additional file 1. Under valgus injury mechanisms, the optimal threshold for LPD to predict LM posterior horn tears was 7.11 mm (sensitivity 0.84, specificity 0.66). Subgroup analysis by injury mechanism further demonstrated that, under valgus extension injury mechanism, the optimal threshold for LPD to predict LM anterior horn tears was 8.45 mm (sensitivity 0.74, specificity 0.86). Under valgus flexion injury mechanism, the optimal threshold for LPD to predict LM posterior horn tears was 7.18 mm (sensitivity 0.76, specificity 0.83).

### Characteristics of TPFs combined with soft-tissue injuries under other injury mechanisms

This study included 80 cases of varus TPFs. And the study demonstrated a high overall prevalence (88.7%) of combined ligamentous and meniscal injuries in varus TPFs, with notable predominance of ACL injuries, LCL injuries, and meniscal tears (particularly the medial meniscus [MM]). Varus flexion TPFs were associated with a higher risk of ACL injuries (82.5%), LCL injuries (65.0%), and meniscal tears (70.0%), while varus hyperextension TPFs showed a higher prevalence of PCL injuries (56.3%; Additional file 1: Table S7).

## Discussion

This study systematically characterized the characteristics of TPFs with concomitant knee soft-tissue injuries involving menisci and ligaments under different injury mechanisms. The findings demonstrated that LPD and LPW were positively correlated with the risk of LM tear under valgus injury mechanisms. On the basis of the valgus classification system for TPFs, LPD emerged as a reliable predictor of fracture combined with LM tears, exhibiting superior diagnostic efficacy compared with LPW, while LPW served as a supplementary diagnostic indicator. Additionally, valgus hyperextension TPFs frequently involved MCL injuries. Extended analysis revealed elevated incidence rates of meniscal tears (particularly medial meniscus), ACL injuries, or LCL injuries in varus-flexion-type fractures, whereas varus hyperextension fractures were more commonly associated with PCL damage.

This study evaluated the association between preoperative CT imaging parameters and LM tears in valgus TPFs. In contrast to previous investigations, this study specifically assessed the predictive capacity of LPD and LPW for LM tears across various valgus injury subtypes. Both LPD and LPW demonstrated significant positive correlations with LM tear risk. Under the overall valgus injury mechanism, a positivity rate of 84% for LM posterior horn tears was observed when LPD > 7.11 mm. Further analysis demonstrated that, in the valgus extension subtype, the positivity rate of LM anterior horn tears was 74% when LPD > 8.45 mm, while in valgus flexion subtype, the positivity rate of LM posterior horn tears was 76% when LPD > 7.18 mm. Existing literature predominantly used the Schatzker classification to analyze the correlation between imaging parameters and LM injuries. In a retrospective study including 62 patients with Schatzker II-type TPFs, Gardner et al. reported an 83% LM injury rate in Schatzker II TPFs with LPD > 6 mm and LPW > 5 mm [8]. Durakbasa et al. identified an elevated risk of LM tear with LPD ≥ 14 mm or LPW ≥ 10 mm [12]. However, above findings were based on X-ray examinations, which have two-dimensional limitations. Ringus et al. measured the LPD values based on CT images and found an eightfold increased risk of LM tear when LPD ≥ 10 mm [22]. Similarly, Liu et al. retrospectively analyzed the imaging data of 60 patients with Schatzker IV-C TPFs and proposed vigilance for LM injuries when LPD > 8.40 mm or LPW > 7.90 mm. [23]. Additionally, Chang et al. [3] reported heightened LM injury risks when the articular depression exceeded 6.3 mm, while Pu et al. [10] determined optimal predictive cut-off values of 7.9 mm for LPD and 7.5 mm for LPW. While these findings align with our observations, the difference is that our study analyzed TPFs on the basis of the injury mechanisms and further elaborated the relationship of LPD and LPW with the anatomical localization of LM tears.

This study identified elevated incidences of meniscal tears, ACL injuries, or LCL injuries in varus flexion TPFs. Luo et al. retrospectively analyzed 67 cases of varus flexion TPFs, identifying a high incidence of avulsion fractures at the ACL insertion [2]. This injury pattern predominantly aligns with Schatzker IV, V, or VI classifications. Yan et al. [24] reported high intra-articular soft-tissue injury rates in Schatzker IV fractures, particularly involving LCL injuries, ACL injuries, and meniscal tears. Peng et al. [25] further established that Schatzker IV fractures involving the posterolateral column were significant correlated with LM and ACL injuries. These findings align with our observational data. Notably, varus hyperextension TPFs were frequently combined with PCL injuries, while valgus hyperextension TPFs showed propensity for MCL involvement. Research indicated that hyperextension TPFs were associated with an increased risk of concomitant ligamentous injuries and less favorable functional outcomes compared with other fracture patterns [26, 27]. Bennet et al. [28] and Yoo et al. [29] further confirmed the association between varus hyperextension TPFs and PCL injuries. And Jiang et al. noted that hyperextension TPFs involving the anteromedial or anterolateral columns alone were prone to combine with “diagonal” injury (MCL or posterolateral complex), particularly when tibial plateau posterior slope angle exceeded 10° [30]. On the basis of these findings, we emphasize the clinical necessity for preoperative MRI evaluation of soft-tissue integrity in both varus flexion and hyperextension TPFs to reduce postoperative complications.

The prediction of soft-tissue injuries involving menisci and ligaments based on injury mechanisms predominantly follows the “diagonal principle.” The side contralateral to the primary site of the fracture (tension side) is usually associated with ligament injury, whereas the side ipsilateral to the fracture site (stress side) is more likely to be associated with meniscus injury [2, 31, 32]. Under the varus flexion injury mechanism, fracture lines predominantly localize to the medial plateau, with collapse often observed in the posterolateral region. This collapse induces anterior displacement of the tibia relative to the femoral condyles, resulting in ACL injury. Concurrently, impaction between the medial femoral condyle and the medial plateau leads to medial meniscal damage. As injury forces intensify, fractures may extend to the tibial intercondylar eminence or lateral plateau, potentially accompanied by LM injuries. Furthermore, during knee flexion, bony stability is reduced and knee stability is largely dependent on soft tissue [33]. Under varus and rotational stresses, the LCL undergoes substantial shear and tensile forces, which leads to LCL injuries [34, 35]. In contrast, under valgus injury mechanisms, the fracture mainly involves the lateral plateau, and the tension side is located medially. Moreover, LCL injuries may arise more from shear forces rather than compressive loading, which explains the limited predictive utility of LPD and LPW for LCL injury assessment.

This study investigated the association between valgus TPFs and concomitant soft-tissue injuries. We not only analyzed the predictive capacity of LPD and LPW for LM tears under various valgus injury mechanisms but also explored the correlation between these parameters and LM tear localization. Additionally, we conducted a systematic study on the characteristics of TPFs combined with soft-tissue injuries involving menisci and ligaments under other injury mechanisms. The present study has the following limitations. First, the retrospective design, limited sample size, and single-center data collection may introduce selection bias. Second, the rarity of valgus hyperextension fractures restricted statistical power to evaluate the correlations of LPD and LPW with LM injuries in this subgroup. Third, the small sample size of LM body tears precluded investigation of the correlation of LPD and LPW with this tear site. Furthermore, the measurements of LPD and LPW were performed by a single senior physician without repeated measurements; therefore, intraobserver and interobserver reliability was not assessed. This constitutes a methodological limitation of the present research. Future studies should employ repeated measurements and multiple observers to further validate the reliability of these measurements.

The findings of this study offer tangible guidance for clinical decision-making. The identified LPD thresholds (7.11 mm for overall valgus, 8.45 mm for valgus extension, 7.18 mm for valgus flexion) can serve as valuable preoperative red flags. When CT scans reveal depression beyond these thresholds, surgeons should maintain a high index of suspicion for concomitant lateral meniscal tears, particularly of the posterior horn in overall and valgus flexion patterns, and the anterior horn in valgus extension injuries. This awareness can prompt more meticulous preoperative planning, such as ensuring the availability of arthroscopic equipment and meniscal repair kits in the operating room, and conducting a more thorough intra-articular assessment during surgery, potentially improving the rates of meniscal preservation and patient outcomes. Building upon this work, several avenues warrant further exploration. First, the predictive thresholds identified herein require external validation in larger, multicenter prospective cohorts to confirm their generalizability. Second, future studies could investigate the biomechanical mechanisms underlying the differential meniscal tear patterns associated with various injury mechanisms using finite element analysis. Third, integrating these imaging parameters with clinical variables (e.g., age, body mass index) into a multivariable predictive model or even an artificial intelligence algorithm could enhance the accuracy of preoperatively predicting soft-tissue injuries involving menisci and ligaments. Lastly, long-term outcome studies are needed to determine whether intervention guided by these thresholds ultimately leads to improved functional results and a reduced risk of post-traumatic osteoarthritis. However, it should be noted that the LPD thresholds and their predictive value proposed in this study are preliminary, exploratory findings and still require prospective validation in large-scale, multicenter studies.

## Conclusions

LPD and LPW positively correlate with the risk of LM tear under valgus injury mechanisms. Among these parameters, LPD serves as a reliable predictor of fractures combined with LM tears, demonstrating superior diagnostic efficacy compared with LPW. Specifically, under the overall valgus injury mechanism, the possibility of LM tears (particularly posterior horn tears) should be guarded against when LPD exceeds 7.11 mm. In the valgus extension subtype, the possibility of LM tears (especially the anterior horn tears) should be highly suspected when LPD exceeds 8.45 mm. And in the valgus flexion subtype, an LPD exceeding 7.18 mm should prompt evaluation for LM tears, particularly those affecting the posterior horn.

## Supplementary Information

**Additional file 1.**

## Data Availability

All the data in the study are available from the corresponding author on reasonable request.

## Abbreviations

ACL: Anterior cruciate ligament
PCL: Posterior cruciate ligament
MCL: Medial collateral ligament
LCL: Lateral collateral ligament
LM: Lateral meniscus
MM: Medial meniscus
LPD: Lateral plateau depression
LPW: Lateral plateau widening
ROC: Receiver operating characteristic
AUC: Area under curve
OR: Odds ratio
CI: Confidence interval
