# Supplementary material for: A study on the association between tibial plateau fractures and intra-articular soft-tissue injuries under valgus injury mechanisms
Source: J Orthop Traumatol. 2026 Apr 25;27:29. doi: 10.1186/s10195-026-00927-5 (PMC13247109; doi:10.1186/s10195-026-00927-5)
Supplement: Supplementary file 1 — Additional file 1. [file 10195_2026_927_MOESM1_ESM.docx]

**Supplementary Information**

Manuscript: **A study on the association between tibial plateau fractures and soft tissue injuries under valgus injury mechanisms**

Authors: Shuo Duan, Tongtong Zhu, Shuaishuai Wang, Minglei Zhang *

* Correspondence: Minglei Zhang

E-mail: [zml669@jlu.edu.cn](mailto:zml669@jlu.edu.cn)

**Supplementary Figure 1. CT images of tibial plateau fractures with different injury mechanisms. (A) represents hyperextension-valgus, (B) represents hyperextension-varus, (C) represents extension-valgus, (D) represents extension-varus, (E) represents flexion-valgus, and (F) represents flexion-valgus**


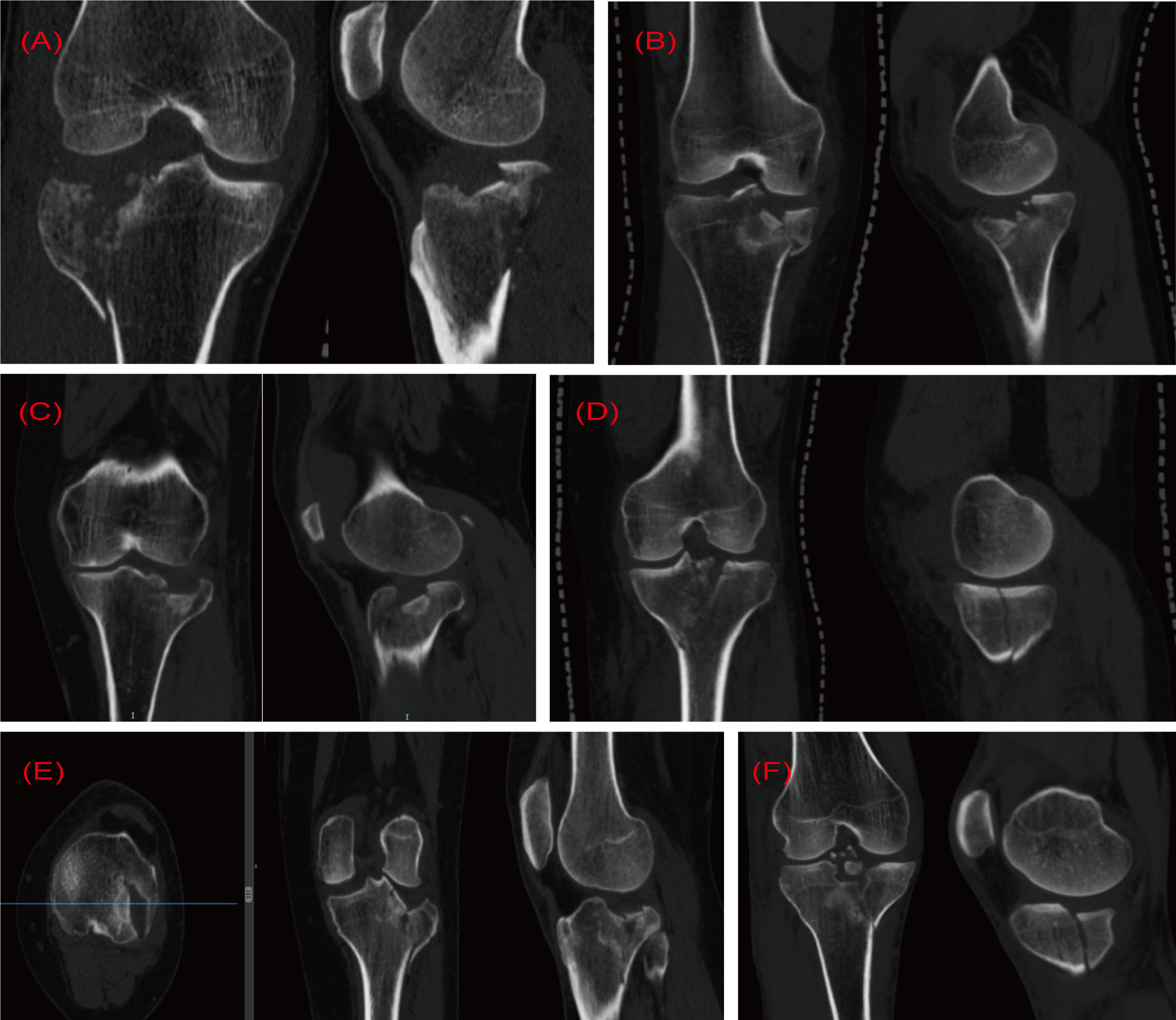


**Supplementary Figure 2. Flowchart for inclusion of participants**


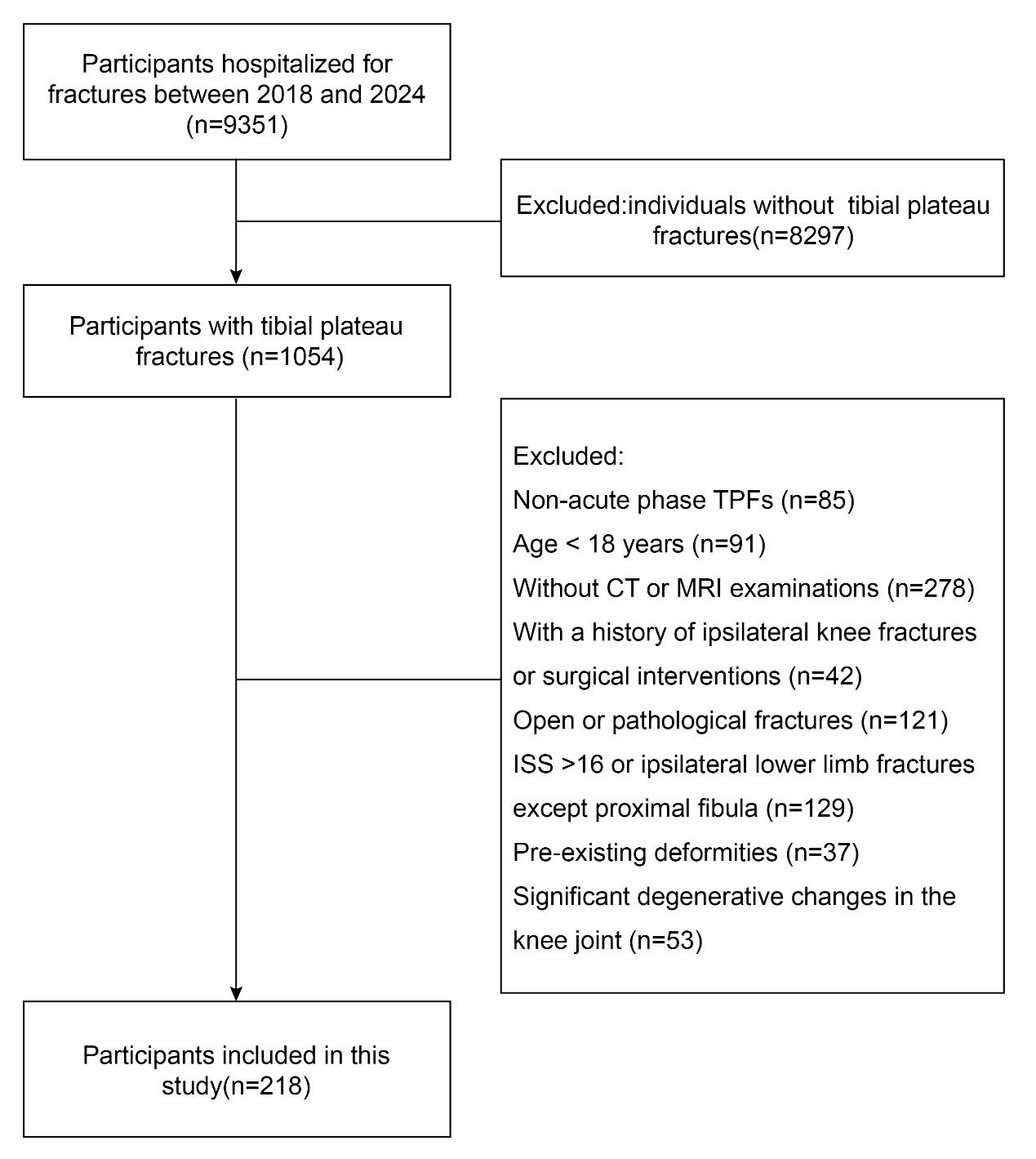


**Supplementary Figure 3. Characteristics of the frequency distribution of injury mechanisms**


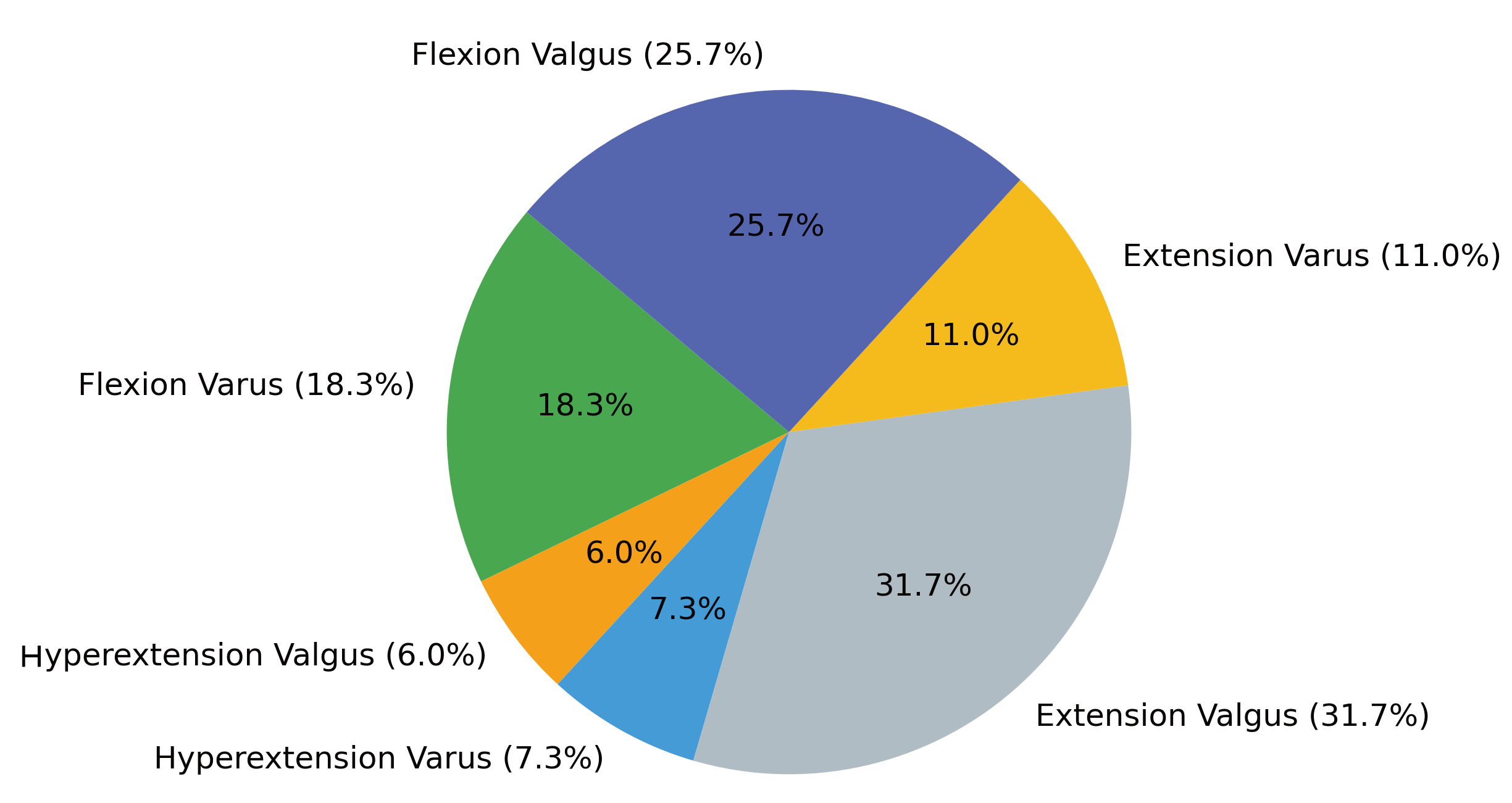


**Supplementary Figure 4. Frequency distribution of meniscal tear locations. Blue indicates medial meniscus, orange indicates lateral meniscus**


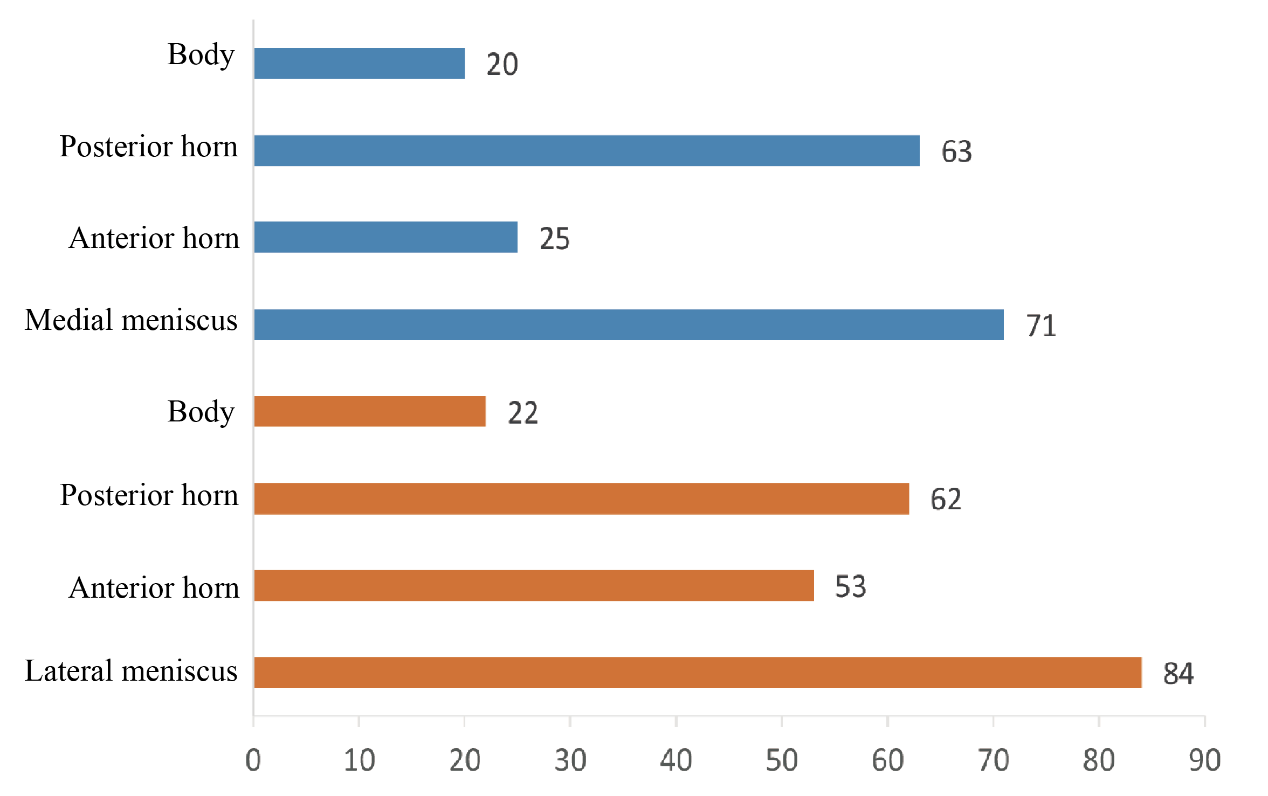


**Supplementary Figure 5. ROC curves of LPD and LPW predicting LM tear location for all valgus fractures.** **The left image represents the meniscus anterior horn and the right image represents the** **meniscus posterior horn**
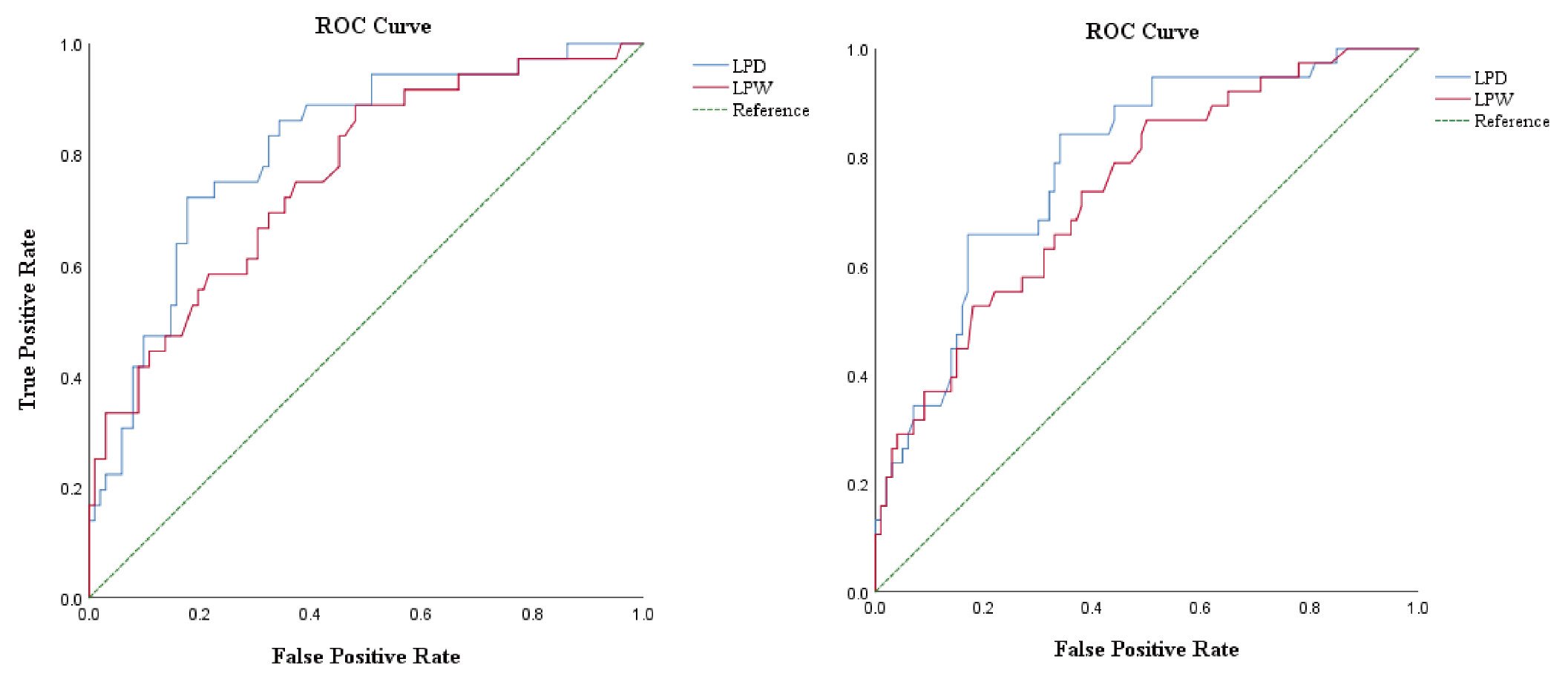


**Supplementary Figure 6. ROC curves of LPD and LPW predicting LM tear location for extension-valgus fractures.** **The left image represents the meniscus anterior horn and the right image represents the** **meniscus posterior horn**


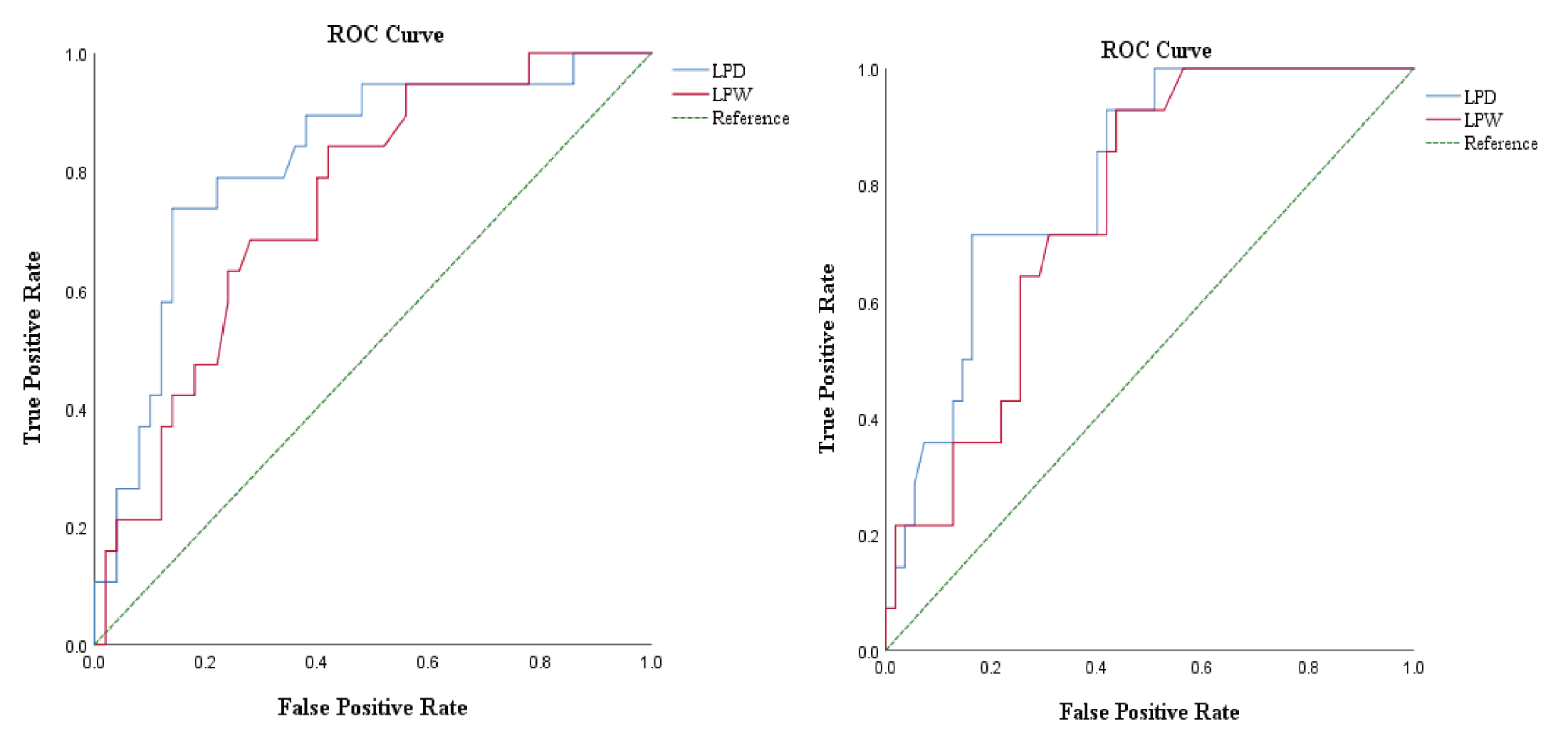


**Supplementary Figure 7. ROC curves of LPD and LPW predicting LM tear location for flexion-valgus fractures.** **The left image represents the meniscus anterior horn and the right image represents the** **meniscus posterior horn**
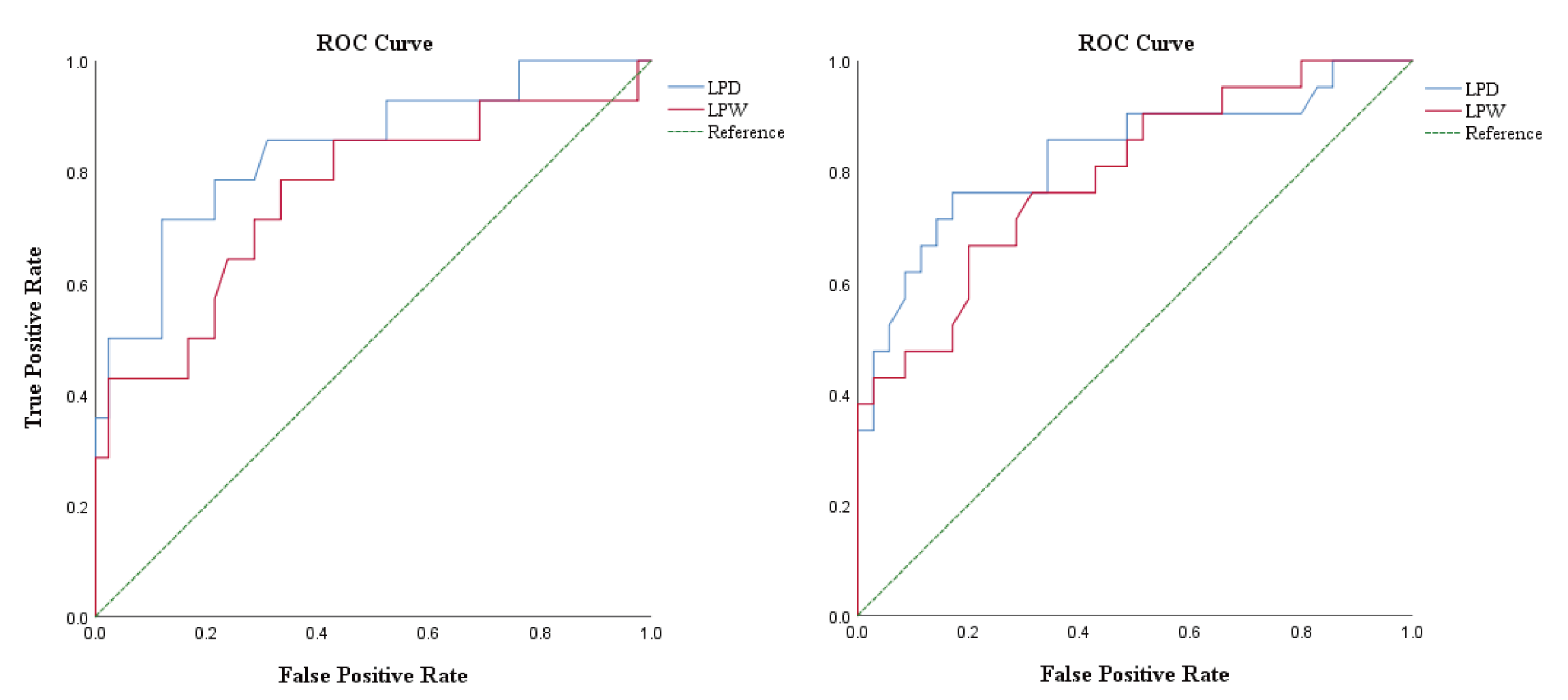


**Supplementary Table 1. Comparison of imaging parameters under different valgus injury mechanisms**

| **Parameters（mm）** | **LM tear** | **Without LM tear** | ***P* value** | **MCL injury** | **Without MCL injury** | ***P* value** |
| --- | --- | --- | --- | --- | --- | --- |
| Valgus |  |  |  |  |  |  |
| LPD | 8.65(7.66, 10.78) | 4.92(3.85, 7.81) | <0.001 | 7.39(4.39. 9.70) | 6.85(4.37, 8.64) | 0.480 |
| LPW | 4.95(3.28, 6.42) | 3.14(2.21, 4.50) | <0.001 | 3.74(2.57, 5.24) | 3.50(2.51, 5.13) | 0.602 |
| Hyperextension  valgus |  |  |  |  |  |  |
| LPD | 7.72(7.31, 9.31) | 6.59(3.80, 13.97) | 0.604 | 8.48(5.35, 11.66) | 8.96(3.80, 16.79) | 0.833 |
| LPW | 7.37(3.80, 10.76) | 3.76(2.05, 4.89) | 0.076 | 3.83(2.58, 8.38) | 4.75(2.05, 5.24) | 0.724 |
| Extension  valgus |  |  |  |  |  |  |
| LPD | 8.64(7.76, 10.91) | 4.56(3.91, 7.97) | <0.001 | 7.30(4.40, 8.80) | 7.81(4.22, 8.64) | 0.832 |
| LPW | 5.12(4.20, 6.12) | 3.50(2.62, 4.78) | <0.001 | 4.46(3.00, 5.45) | 3.95(2.94, 5.43) | 0.817 |
| Flexion valgus |  |  |  |  |  |  |
| LPD | 8.65(6.57, 11.15) | 5.13(3.76, 6.74) | <0.001 | 6.37(3.97, 10.98) | 6.66(4.71, 8.40) | 0.902 |
| LPW | 3.73(2.79, 6.34) | 2.45(2.15, 3.26) | 0.001 | 3.01(2.17, 3.97) | 3.13(2.25, 4.54) | 0.813 |

**Supplementary Table 2. Logistic regression analyses of LPD and LPW with ligaments injuries**

| **Variable** | **ACL** | | **PCL** | | **LCL** | | | **MCL** | |
| --- | --- | --- | --- | --- | --- | --- | --- | --- | --- |
|  | **OR**  **(95%CI)** | ***P* value** | **OR**  **(95%CI)** | ***P* value** | | **OR**  **(95%CI)** | ***P* value** | **OR**  **(95%CI)** | ***P* value** |
| LPD | 0.989(0.909,1.077) | 0.802 | 0.972(0.886,1.067) | 0.556 | | 1.127(1.027,1.238) | 0.012 | 1.051(0.967,1.142) | 0.241 |
| LPW | 0.996(0.829,1.196) | 0.964 | 0.985(0.814,1.191) | 0.873 | | 1.219(1.012,1.168) | 0.037 | 1.097(0.916,1.314) | 0.314 |

**Supplementary Table 3. AUC of LPD and LPW predicting the location of lateral meniscus tears**

| **Variable** | **Meniscus anterior horn** | |  | **Meniscus posterior horn** | |
| --- | --- | --- | --- | --- | --- |
|  | **AUC** | **95%CI** |  | **AUC** | **95%CI** |
| Valgus |  |  |  |  |  |
| LPD | 0.812 | 0.732, 0.892 |  | 0.789 | 0.708, 0.871 |
| LPW | 0.762 | 0.671, 0.853 |  | 0.741 | 0.650, 0.831 |
| Extension  valgus |  |  |  |  |  |
| LPD | 0.818 | 0.706, 0.931 |  | 0.810 | 0.699, 0.920 |
| LPW | 0.745 | 0.623, 0.866 |  | 0.758 | 0.639, 0.877 |
| Flexion valgus |  |  |  |  |  |
| LPD | 0.843 | 0.715, 0.970 |  | 0.830 | 0.710, 0.950 |
| LPW | 0.759 | 0.599, 0.920 |  | 0.793 | 0.671, 0.915 |

**Supplementary Table 4. Optimal cut-off values for LPD and LPW predicting the location of LM tears for all valgus injury mechanisms**

| **Variable** | **Youden index** | **Optimal threshold** | **Sensitivity** | **Specificity** |
| --- | --- | --- | --- | --- |
| LPD |  |  |  |  |
| Anterior horn | 0.54 | 8.45 | 0.72 | 0.82 |
| Posterior horn | 0.50 | 7.11 | 0.84 | 0.66 |
| LPW |  |  |  |  |
| Anterior horn | 0.41 | 3.19 | 0.89 | 0.52 |
| Posterior horn | 0.37 | 3.16 | 0.87 | 0.50 |

**Supplementary Table 5**. **Optimal cut-off values for LPD and LPW predicting the location of LM tears for extension-valgus injury mechanisms**

| **Variable** | **Youden index** | **Optimal threshold** | **Sensitivity** | **Specificity** |
| --- | --- | --- | --- | --- |
| LPD |  |  |  |  |
| Anterior horn | 0.60 | 8.45 | 0.74 | 0.86 |
| Posterior horn | 0.55 | 8.52 | 0.71 | 0.84 |
| LPW |  |  |  |  |
| Anterior horn | 0.42 | 4.05 | 0.84 | 0.58 |
| Posterior horn | 0.49 | 4.06 | 0.93 | 0.56 |

**Supplementary Table 6**. **Optimal cut-off values for LPD and LPW predicting the location of LM tears for flexion-valgus injury mechanisms**

| **Variable** | **Youden index** | **Optimal threshold** | **Sensitivity** | **Specificity** |
| --- | --- | --- | --- | --- |
| LPD |  |  |  |  |
| Anterior horn | 0.59 | 8.61 | 0.71 | 0.88 |
| Posterior horn | 0.59 | 7.18 | 0.76 | 0.83 |
| LPW |  |  |  |  |
| Anterior horn | 0.45 | 3.19 | 0.79 | 0.67 |
| Posterior horn | 0.47 | 3.37 | 0.67 | 0.80 |

**Supplementary Table 7. Association between tibial plateau fractures and soft tissue injuries under varus injury mechanisms**

| **Variable** | **Total** | **Injury mechanism** | | | ***P* value** |
| --- | --- | --- | --- | --- | --- |
|  |  | **Hyperextension varus** | **Extension varus** | **Flexion varus** |  |
| Meniscus tear | 43(53.8) | 5(31.2) | 10(41.7) | 28(70.0) | 0.012 |
| LM tear | 29(36.3) | 2(12.5) | 9(37.5) | 18(45.0) | 0.073 |
| MM tear | 37(46.3) | 5(31.3) | 10(41.7) | 22(55.0) | 0.237 |
| ACL injury | 57(71.3) | 6(37.5) | 18(75.0) | 33(82.5) | 0.003 |
| PCL injury | 36(45.0) | 9(56.3) | 8(33.3) | 19(47.5) | 0.326 |
| LCL injury | 40(50.0) | 7(43.8) | 7(25.9) | 26(65.0) | 0.018 |
| MCL injury | 23(28.8) | 3(18.8) | 6(25.0) | 14(35.0) | 0.426 |
